# The Frequency and Perceived Effectiveness of Pain Self-Management Strategies Used by Individuals With Migraine

**DOI:** 10.1097/jnr.0000000000000429

**Published:** 2021-04-09

**Authors:** Hao-Yuan CHANG, Chih-Chao YANG, Mark P. JENSEN, Yeur-Hur LAI

**Affiliations:** 1 PhD, RN, Assistant Professor, School of Nursing, College of Medicine, National Taiwan University, and Adjunct Supervisor, Department of Nursing, National Taiwan University Hospital, Taiwan, ROC; 2 MD, Attending Physician, Department of Neurology, National Taiwan University Hospital, Taiwan, ROC; 3 PhD, Professor, Department of Rehabilitation Medicine, University of Washington, Seattle, WA, USA; 4 PhD, RN, FAAN, Professor, School of Nursing, College of Medicine, National Taiwan University, and Director, Department of Nursing, National Taiwan University Cancer Center, Taipei, Taiwan, ROC.

**Keywords:** migraine, pain management, self-management, perceived effectiveness

## Abstract

**Background:**

Migraine is ranked among the most important causes of disability worldwide. Some effective migraine treatments have been identified. However, little is known regarding the treatment strategies used by patients with migraine to manage pain or their efficacy.

**Purpose:**

This study was designed to (a) investigate the pain management strategies used by migraineurs and their perceived effectiveness and (b) evaluate the association between the number of strategies used and their overall perceived effectiveness.

**Methods:**

A cross-sectional design with consecutive sampling was used in a medical center in Taiwan. Individuals with migraine (*N* = 174) completed self-administered questionnaires and in-depth interviews to assess the frequency and perceived effectiveness of a variety of pain management strategies.

**Results:**

Most participants reported using prescription medications (56%) and over-the-counter medications (51%), which were rated as having good efficacy rates of 78% and 81%, respectively. Traditional Chinese medicine (17%) and folk remedies (13%) were used less frequently and rated as relatively less effective at 65% and 48%, respectively. About half (47%) reported using more than one pain management strategy. Significantly more of those who reported using multiple pain management strategies reported at least “some effect” than those who reported using one strategy only (73% vs. 27%, *p* = .001).

**Conclusions:**

Prescription medications showed good usage rate and good perceived efficacy. However, about half of the participants used multiple pain management strategies, supporting the need for further research to evaluate the efficacy of combination treatments and to identify those combinations that may have the most additive and/or synergistic effects. Furthermore, the findings indicate that continued use of medications for migraine management is appropriate for many individuals because of the relatively high rates of perceived efficacy for this strategy found in this study.

## Introduction

Migraine is one of the most important causes of disability worldwide, affecting estimated 1.04 billion people worldwide in 2016 (Stovner et al., 2018). Moreover, migraine-related disability and missed workdays cause significant human downtime (an estimated 45.1 million years of life lived with disability globally in 2016; Stovner et al., 2018) and a significant financial cost (an estimated loss of $13 billion dollars a year in the United States; Hu et al., 1999; Silberstein & Marmura, 2015). Headache is a universal health problem, even in nurse populations (Ko et al., 2018), further supporting the importance of appropriate migraine management.

Research has identified a number of effective migraine treatments, including abortive medications (e.g., triptans, nonsteroidal anti-inflammatory drugs; Dodick, 2018; Macone & Perloff, 2017) and preventive medications (e.g., beta-blockers, calcium channel blockers; Mayans & Walling, 2018). However, little is known regarding how patients with migraine actually manage their pain and how they perceive the efficacy of their pain management/treatment strategies. In addition, little is known regarding the association between the number of pain management strategies used and overall effectiveness.

Research to address these knowledge gaps would clarify the extent to which migraineurs use the management strategies that have been identified as effective in the literature. Such research could potentially identify new or understudied management approaches that warrant additional research to evaluate their efficacy. Moreover, knowledge in this area could provide insights for clinical professionals to facilitate their consulting with patients regarding management strategies that may be most effective.

Given these considerations, the purposes of this study were to (a) investigate the frequency of use and perceived efficacy of different pain management strategies actually used by migraineurs seeking treatment by physicians and (b) evaluate the association between the number of pain management strategies used and overall effectiveness.

We hypothesized that pain management strategies with the most solid empirical support (e.g., medications) would emerge as being used most frequently and also rated as the most effective, relative to other migraine pain management strategies. However, we also sought to determine if there were additional pain management strategies used that were rated as being effective by at least some individuals with migraine and thus potentially worthy of further empirical study. Moreover, considering the potential synergistic effects of different treatments when used in combination, we also hypothesized that the number of pain management strategies adopted by migraineurs would relate positively to overall effectiveness.

## Methods

### Design

A cross-sectional design was adopted to address the study aims. Consecutive sampling was adopted to maximize sample representation. Consecutive sampling refers to the sampling method in which all members of an identified population are approached and invited to participate over a fixed period (Polit & Beck, 2017).

### Setting and Participants

Patients were diagnosed as having migraine by four board-certified neurologists based on International Headache Society criteria. We recruited participants from nine different clinics (seven neurology clinics and two general medicine clinics) of a large medical center in Taiwan from September 2015 through July 2016. Inclusion criteria were (a) being 20–65 years old and (b) having a migraine diagnosis based on International Headache Society criteria, including migraine without aura (International Classification of Headache Disorders [ICHD] Code 1.1) and migraine with typical aura (ICHD 1.2.1). Exclusion criteria were being diagnosed with (a) a rare migraine type, basilar-type migraine (ICHD Code 1.2.2), including hemiplegic migraine (ICHD Code 1.2.3), retinal migraine, and other migraine types (ICHD Codes 1.2.4–1.2.6 and 1.3–1.5) or (b) a mixed headache type, for example, combination of migraine and tension-type headache. Medication overuse headache (ICHD Code 8.2), defined as a headache attributed to a substance or its withdrawal, was also excluded.

Patients who met the inclusion/exclusion criteria were referred to our research team. When these potential participants returned to the clinic for a subsequent visit, a research assistant described the study to them and invited them to participate. Those who agreed to participate were asked to sign the informed consent form before any data were collected.

The estimated sample size was calculated using G*Power Version 3.1.9.2. For an effect size *f* = .25, alpha = .05, power = .80, and number of groups = 3, the estimated sample size was set at a minimum of 159. From September 2015 through July 2016, we approached 214 potential participation patients who met the inclusion criteria. Thirteen (6%) declined to participate, 25 (12%) did not return to the clinic again, and two (1%) withdrew from the study after completing the interview. This left valid information from 174 participants (81% of those who were eligible) in the final data set.

### Ethical Considerations

All of the study procedures were approved by the institutional review board at National Taiwan University Hospital (201505065RIND). Signed informed consent was obtained from participants before data collection. Data were collected by three trained research assistants who followed a standardized assessment procedure.

### Measures

The data presented in this article were collected as part of a survey study on a group of patients with migraine that used semistructured questionnaires and in-depth interviews to survey patient perspectives (Polit & Beck, 2017). Domains assessed included pain management strategies used, the perceived effectiveness of these strategies, pain intensity, migraine-related disability, and demographic information. The Migraine-Specific Quality of Life Chinese Version 2.1 (MSQv2.1-C) was also administered to this group, and the findings related this measure have already been reported (Chang et al., 2019). No overlap in study aims exist between Chang et al. and this study.

#### Pain management strategies and perceived effectiveness

Participants answered questions regarding “headache pain management” and perceived effectiveness, including “pain relief” and “effect duration.” The first, open-ended question was “What pain management strategies have you ever used for your migraine?” Participants were then asked to describe each pain management strategy they had used in as much detail as possible. Each strategy was then was coded into specific pain management categories, including prescription medicine, over-the-counter (OTC) medicine, traditional Chinese medicine (TCM), folk remedy, and other management strategies.

The second question was “Please circle the percentage amount to indicate how much pain relief you obtained using this treatment.” This question assessed the amount of pain relief that the participant achieved using each pain management strategy. The respondent could circle one of 11 response options along a graded scale ranging from 0% (*no relief*) to 100% (*total relief*). Some of the participants asked if they could indicate a pain relief level between two response options (e.g., 55% or 65%), which was allowed.

The third question was “How long did the pain relief last?” Participants were asked to choose one response for each pain management strategy used on a 4-point Likert scale: 1 = *no effect*, 2 = *less than 1 day*, 3 = *more than 1 day*, and 4 = *I have not had a migraine episode since this treatment.* For prescription medications, “treatment” reflected the dose and frequency of use as, although the immediate effects of most prescription medications are short-lived (i.e., less than 24 hours), treatment often lasts for an extended period.

#### Demographic and pain-related data

Basic demographic information (age, education level, and marital status) were provided by the participants for descriptive purposes. Average pain intensity during the past 3 months and worst pain intensity in the last migraine episode were measured using a 0–10 numerical rating scale, with 0 = *no pain at all* and 10 = *worst pain I can imagine.*

Migraine-related disability was measured using the Migraine Disability Assessment (MIDAS; Hung et al., 2006; Stewart et al., 1999). The MIDAS measures three aspects of daily life: employment (work/school), household work, and nonwork activities. On the MIDAS, respondents are asked to indicate the number of days that five different activities were limited by migraine. Total possible MIDAS scores range from 0 to 270, with the result used to classify respondents into four grades of migraine-related disability: (a) 0–5, little or no disability; (b) 6–10, mild disability; (c) 11–20, moderate disability; and (d) ≥ 21, severe disability.

The MIDAS was developed by Stewart et al. (1999) and has shown good reliability and validity, with a 21-day test–retest reliability of .67–.73 for the items and .84 for the total scale. Internal consistency reliability (Cronbach's alpha) was found to be .83 and .79, respectively, in the original scale development sample and in this study. MIDAS scores have been shown to differ between migraineurs and nonmigraineurs, supporting the discriminant validity of the measure (Stewart et al., 1999). The MIDAS has been translated from English into other languages, including traditional Chinese (Hung et al., 2006), Japanese (Iigaya et al., 2003), and Turkish (Ertas et al., 2004). All of the translated versions of this scale have shown good reliability and validity.

### Data Analysis

Descriptive statistics were first computed with the demographic, migraine/pain history, and disability measures to describe the sample. Next, the frequency, percentage, means, and standard deviations of the responses to the pain management use and effectiveness questions were calculated. Chi-square analysis was used to examine the association between the number of strategies used and overall effectiveness. Analysis of variance using Scheffe's post hoc test was used to examine the association between attack severity and usage of multiple pain management strategies. IBM SPSS Version 21.0 (IBM, Armonk, NY, USA) was used for all statistical analyses, and the level of significance was set as .05.

## Results

### Description of the Participants

Demographic and pain-related data for the sample are shown in Table 1. The average age was 38.5 years (*SD* = 11.8), and most held a college degree (66%) and were married (53%). The mean worst pain intensity score for the most recent migraine episode was 6.3/10 (*SD* = 2.1), and the mean average pain intensity score of migraine headaches during the past 3 months was 5.5/10 (*SD* = 2.1). The plurality of disability grade was “minimal or infrequent” (48%).

**Table 1.** Demographics and Pain-Related Data of the Participants (*N* = 174)

Variable	*n*	%	Mean	*SD*	Range
Age (years)			38.5	11.8	20–65
20–30	49	28			
31–40	57	33			
41–50	35	20			
51–60	26	15			
61–65	6	3			
Missing (refuse to provide)	1	1			
Body mass index			22.6	3.9	15.0–40.1
Education					
High school or less	32	18			
College	115	66			
Graduate school	27	16			
Marital status					
Unmarried	75	43			
Married	92	53			
Ever married	7	4			
Average pain intensity			5.5	2.1	0–10
Mild (1–4)	55	32			
Moderate (5–6)	59	34			
Severe (7–10)	60	34			
Worst pain intensity			6.3	2.1	1–10
Mild (1–4)	33	20			
Moderate (5–6)	47	28			
Severe (7–10)	85	52			
Disability (MIDAS)			16.0	33.3	0–270
Minimal or infrequent (0–5)	84	48			
Mild (6–10)	32	18			
Moderate (11–20)	27	16			
Severe (21 or above)	31	18			
Number of pain management strategies used					
One	93	53			
Two	48	28			
More than three	33	19			

***Note.*** MIDAS = Migraine Disability Assessment.

### Use and Perceived Effectiveness of Pain Management Strategies

As shown in Table 2, most participants used physician-prescribed medications (56%, e.g., Imigran, Cafergot, Inderal), 51% used OTC medications (e.g., acetaminophen, acetaminophen plus caffeine, caffeine plus ergotamine, or nonsteroidal anti-inflammatory drugs), 17% used TCM, and 13% used a folk remedy (e.g., massage or “gua sha,” an instrument-assisted massage that induces cutaneous petechiae). During the interviews, the participants indicated that gua sha was usually used during acute pain episodes. Some of the participants indicated that they were given gua sha massages by their friends or relatives, and some reported that they were given gua sha massages by masseurs. Other, significantly less-frequently used strategies included rest/sleep, coffee, and essential oils applied to the body.

**Table 2.** Pain Management and Relief Ratings Reported by the Participants (*N* = 174)

Pain Management	*n*	%	Pain Relief			Effective Rate ^a^ (%)	Effective Duration									
							No effect		≤ 1 Day		> 1 Day		No Attack		Missing ^b^	
			*M*	*SD*	[Median, Mode]		*n*	%	*n*	%	*n*	%	*n*	%	*n*	%
Prescription medicine	98	56	65	31.0	[70, 100]	78	8	8	13	13	49	50	13	13	15	15
Abortive ^c^	80	46	69	27.6	[70, 100]	82	5	6	11	14	40	50	11	14	13	16
Rescue ^d^	24	14	53	34.8	[60, 0 & 80]	67	3	13	2	8	11	46	3	13	5	21
Preventive ^e^	29	17	66	32.3	[65, 100]	80	1	3	5	17	10	34	4	14	9	31
Over-the-counter medicine ^f^	89	51	70	31.0	[80, 100]	81	9	10	20	22	32	36	23	26	5	6
Acetaminophen	36	21	64	32.2	[70, 100]	79	4	11	6	17	13	36	10	28	3	8
Analgesics (unknown)	23	13	70	35.3	[80, 100]	73	3	13	7	30	7	30	6	26	–	–
NSAIDs	22	13	80	20.2	[85, 100]	90	–	–	5	23	9	41	7	32	1	5
Acetaminophen + caffeine	8	5	52	43.2	[60, 0]	67	2	25	2	25	3	38	–	–	1	13
Traditional Chinese medicine	30	17	52	27.7	[60, 60 & 70]	65	11	37	5	17	6	20	2	7	6	20
Chinese herbs	19	11	49	26.2	[60, 60]	67	10	53	1	5	4	21	1	5	3	16
Acupuncture	7	4	68	26.8	[80, 40 & 80]	60	–	–	3	43	1	14	1	14	2	29
Herbs + acupuncture	4	2	40	36.1	[50, 0 & 50 & 70]	67	1	25	1	25	1	25	–	–	1	25
Folk remedy	22	13	49	32.1	[40, 30]	48	5	23	8	36	5	23	2	9	2	9
Massage	20	11	45	31.0	[40, 30]	42	5	25	7	35	5	25	1	5	2	10
“Gua sha” therapy	4	2	63	28.7	[55, 40]	50	1	25	2	50	–	–	1	25	–	–
Hot packing	1	< 1	30	0.0	[30, 30]	0	–	–	–		1	100	–	–	–	–
Electronic therapy	1	< 1	40	0.0	[40, 40]	0	1	100	–	–	–		–	–	–	–
Other management	32	18	57	33.1	[60, 60 & 100]	64	5	16	8	25	12	38	3	9	4	13
Rest/sleep	11	6	64	36.0	[70, 100]	70	1	9	2	18	4	36	3	27	1	9
Hot coffee	11	6	51	36.7	[60, 0 & 60]	64	2	18	3	27	6	55	–	–	–	–
Essential oil (applied to body)	6	3	26	24.0	[40, 0 & 40]	20	3	50	2	33	1	17	–	–	–	–
Healthy supplement	1	< 1	100	0.0	[100, 100]	100	–	–	–	–	1	100	–	–	–	–
Meditation	1	< 1	90	0.0	[90, 90]	100	–	–	–	–	1	100	–	–	–	–
Pray	1	< 1	70	0.0	[70]	100	–	–	1	100	–	–	–	–	–	–
Emetic by finger	1	< 1	60	0.0	[60]	100	–	–	1	100	–	–	–	–	–	–
Beating head	1	< 1	30	0.0	[30, 30]	0	1	100	–	–	–	–	–	–	–	–

***Note.*** NSAIDs = nonsteroidal anti-inflammatory drugs.

^a^ Treatments that provided a 50% or greater amount of pain relief were defined as effective. Effective rate refers to the proportion of respondents endorsing effective pain relief among those who used the pain management approach; ^b^ Participants who find that a pain management approach is not consistently effective are not able to rate the duration of effect for that approach; ^c^ Abortive (e.g., triptans, ergotamine tartrate plus caffeine, nonsteroidal anti-inflammatory drugs, antiemetics);

^d^ Rescue (e.g., tramadol plus acetaminophen, benzodiazepine, anxiolytics, muscle relaxant, anticoagulants); ^e^ Preventive (e.g., β-blocker, antidepressant, anticonvulsant, calcium channel blockers); ^f^ Over-the-counter medicine refers to the medicine bought from pharmacy.

Of the listed pain management strategies, 8%–37% were reported to be totally ineffective, with prescribed medications reported as ineffective the least often (prescription medications: 8%; OTC medications: 10%; TCM: 37%; folk remedies: 23%; other strategies: 16%). Twenty-five (14%) of the participants reported all of the pain management strategies used as totally ineffective.

Two themes emerged from the interviews: (a) considering both Western and Chinese medicine and (b) managing the headache.

#### Considering both Western and Chinese medicine

The three subthemes that emerged under this theme were (a) uncertainty regarding the effects of treatment, (b) using a combination of Chinese and Western medicine approaches, and (c) using Western medicine as a last resort.

Uncertainty regarding the effects of treatment: Whatever the pain treatment, participants reported uncertainty regarding its effects. For example, “*Painkillers are not always effective*” (Case 69). Because of this uncertainty, the participants sought other pain management strategies for their migraine. For example, “*Panadol Extra is not effective, so I went for Chinese medicine*” (Case 48), “*Traditional Chinese medicine was effective one month ago, but now, it is gone. The effect is not immediate; the effect is uncertain*” (Case 41), and “*I went to seek a physician's help because I found the OTC was not effective for my headache*” (Case 97).

Using a combination of Chinese and Western medicine approaches: Some participants choose to use both Chinese and Western medicine (Cases 56, 98, and 99). For example, “*I take Western medicine in the morning and evening and take Chinese medicine after meals three times a day. If the pain persists, I take OTC medicine*” (Case 56) and “*When it hurts as much as a 2 (Numerical Rating Scales pain scale), I take Western medicine (acetaminophen). I also take Chinese medicine four times a day*” (Case 99).

Using Western medicine as a last resort: The timing of taking pain medicine was polarized. Only seven participants reported that they “*Take medicine as soon as it hurts*” (*n* = 8; Cases 65, 82, 141, 149, 151, 154, 159, and 161), whereas 27 participants reported: “*I take the medicine when I cannot endure the pain*” (*n* = 27; Cases 7, 17, 19, 21, 23–25, 30, 35, 38, 72, 77, 86, 96, 133, 135, 136, 140, 148, 152, 153, 158, 160, 163, 167, 170, and 176). Some reported trying massage first and taking medicine only if the pain persisted (*n* = 5; Cases 22, 148, 153, 160, and 163): “*It hurts, but I need to work. Thus, I took Panadol*” (Case 6).

#### Managing the headache

The four subthemes that emerged under this theme were (a) seeking to identify a “protocol” suitable for their headache, (b) avoiding aggravating factors and triggers, (c) adopting regular prevention, and (d) adopting passive coping.

Seeking to identify a “protocol” suitable for their headache: For example, “*I have coffee every day. When it hurts, I take Naposin as soon as possible, and then sit and do not move. If it still hurts after one hour, I will take Imigran*” (Case 65) and “*If my pain score is only 5, I take Panadol Extra (acetaminophen plus caffeine). If my pain scale is 8, I will take Imigran. If Imigran is not effective, I will take Panadol Extra six hours later*” (Case 18).

Avoiding aggravating factors and triggers: The participants reported avoiding aggravating factors and triggers such as cold environments, high stress, fatigue, cheese products, and pungent smells: “*I try to find the source of my headache such as being in environments with large temperature differences, high stress, or fatigue*” (Case 11), “*I know that my headache will become more severe after drinking coffee*” (Case 10), and “*I avoid cheese products and other strange smells such as smoke, betel nut, and pungent perfume*” (Case 27).

Adopting regular prevention such as drinking coffee every day or taking preventive medication: For example, “*Drinking coffee during the day and ginger tea at night*” (Case 26), “*I take Inderal daily. When I have an attack, I will take Imigran with Paramol, Ibuprofen, or Naposin. Otherwise, I have a cup of coffee every day*” (Case 27), and “*If I do not have a cup of coffee, I will have a headache that day*” (Case 63).

Adopting passive coping: Some participants reported that they do not take medicine, receive massage, or engage in other active management measures but rather wait for improvement: “*I just lie on my bed and wait for relief…because…I think it's useless to take medicine. You know that…during a (migraine) attack I will vomit. Even if I take medicine, I will vomit it out*” (Case 11).

#### Summary of qualitative findings

Many participants viewed Western medication approaches as a last resort. The participants reported holding some concerns regarding the possible side effects of medications (such as the hepatotoxicity of Panadol) and preferred using approaches that do not involve prescription medications (such as massage, coffee, and Chinese medicine) to manage their pain. However, when these approaches did not effectively reduce pain, many then used Western pain medicines because of their perceived high efficacy. Furthermore, the participants often adopted multiple strategies to manage their migraine.

Nearly half (47%) of the participants reported using more than one pain management strategy. These combined treatments and their level of pain relief are listed in Table 3. From the perspective of the participants, “OTC only,” “OTC + other management,” and “OTC + folk remedy (massage) + other management” were rated as the most effective (combined) treatments. Moreover, the efficacy (i.e., pain relief) of “single” pain management strategies (except for TCM) was found to be better than that of combined treatment strategies (Table 3).

**Table 3.** Pain Relief Provided by Each Pain Management Combination, by Ranking (*N* = 174)

Combination	*n*	Mean (%) ^a^	Rank
OTC	30	75	1
OTC + OM	6	69	2
OTC + FR (massage) + OM	2	68	3
FR (massage [2]/gua sha [3])	5	66	4
OTC + FR (massage)	3	63	5
PM	42	62	6
PM + OTC + TCM	8	61	7
OM	11	58	8
PM + OTC	23	55	9
PM + OTC + FR + OM	1	55	10
PM + OTC + TCM + OM	3	54	11
PM + OTC + OM	2	53	12
PM + OTC + FR	5	51	13
PM + OTC + TCM + FR	4	50	14
PM + TCM + FR	3	42	15
PM + TCM	7	41	16
PM + FR + OM	3	41	17
PM + OTC + TCM + FR + OM	1	40	18
PM + OM	3	35	19
PM + FR	5	32	20
OTC + TCM	1	28	21
TCM + OM	1	20	22
None	3	0	23
TCM	2	0	23

***Note.*** OTC = over-the-counter medicine; OM = other management; FR = folk remedy; PM = prescription medicine; TCM = traditional Chinese medicine.

^a^ Mean scores were calculated as the average of the pain relief scores of the pain management combination. For example, for the two participants who chose the combination “OTC + FR (massage) + OM,” one reported pain relief scores for OTC, FR, and OM as 80%, 30%, and 50%, respectively (i.e., average pain relief for this combination = 53%), whereas the other reported pain relief scores for OTC, FR, and OM as 100%, 70%, and 80%, respectively (i.e., average pain relief for this combination = 83%). Thus, the mean score for the combination “OTC + FR (massage) + OM” is 68% (the average of 53% and 83%).

Of those participants who reported using two or more pain management strategies, significantly more reported the effectiveness to be at least “some effect” (73% vs. 27%, χ^2^(2) = 14.6, *p* = .001; Table 4). Use of multiple pain management strategies was associated with attack severity (*F* = 5.94, *p* = .003; Scheffe's post hoc: worst pain intensity 7.3 [three or more pain management strategies] vs. 5.8 [one strategy], *p* = .004).

**Table 4.** Association Between Average Effectiveness and Number of Pain Management Strategies Used

Average Effectiveness ^a^	Number of Pain Management Strategies Used				Total		Chi-Square	*p*
	One		≥ Two					
	*n*	%	*n*	%	*n*	%		
Totally ineffective (0)	17	68	8	32	25	100	14.6 (*df* = 2)	.001
Some effect (1.0–4.9)	11	27	29	73	40	100		
Effective (5.0 or above)	65	60	44	40	109	100		
Total	93	53	81	47	174	100		

**^a^** Average effectiveness (possible range: 0–10) = sum of pain relief score / sum of the number of pain management strategies used.

## Discussion

To the best of our knowledge, this was the first study to examine from the perspective of patients how patients manage their migraine. As hypothesized, prescription and OTC medications were used most often and rated as being most effective. Furthermore, almost half of the participants reported using more than one pain management strategy. These findings have important clinical and research implications.

### Prescription and Over-the-Counter Medications

Triptans are effective in managing migraine for many people (Dodick, 2018; Macone & Perloff, 2017) in terms of providing total pain relief within 2 hours and continued pain relief for at least 24 hours (Lipton et al., 2017). Consistent with this finding, prescription medications, including triptans, were shown in this study to provide better efficacy (a larger proportion of participants reporting them as effective) and effect duration than other migraine pain management strategies. Prescription (65%) and OTC (70%) medications were identified as providing more pain relief than the other three pain management options (49%–57%). These findings suggest that OTC medications may be the most effective pain management approaches used from the patients' perspective.

From the perspective of the participants, “OTC only,” “OTC + other management,” and “OTC + folk remedy (massage) + other management” were rated as the most effective combined treatments. These results may explain why patients with migraine use OTC medication so often. OTC medications are easy to obtain and provide good pain relief. However, for patients experiencing severe levels of migraine pain, the potential of developing medication overuse headache should be considered as a risk of using OTC drugs.

### Traditional Chinese Medicine

TCM treats migraine as a symptom of imbalance in the “Qi” and insufficient blood perfusion in organs (Xiao et al., 2015). TCM doctors typically prescribe Chinese herbs/formulas to balance the Qi, promoting blood circulation and reducing stasis (Xiao et al., 2015). Consistent with research supporting the efficacy of TCM (Luo et al., 2020), the participants in this study rated herbs as being effective more often than not (67%). Thus, the findings in this study concur with the TCM perspective with respect to the perceived efficacy of related treatments.

Acupuncture is primarily used for prevention and has been shown to reduce pain intensity and improve quality of life (Jiang et al., 2018). In this study, the substantial effectiveness of acupuncture was shown to be highly effective (effective rate: 68%), although it was also found that the benefits of acupuncture lasted for less than 1 day in most participants. Thus, the findings suggest an important potential limitation of using acupuncture to manage acute pain attacks and may help explain why so few participants chose this approach (only 6% in our sample). The findings indicate that future acupuncture research should examine the duration of treatment benefits more closely. If the maintenance of benefits is found to be limited, as suggested in this study, research may be needed to identify strategies to enhance the maintenance of gains such as teaching patients to self-administer acupressure using, for example, a self-acupressure pillow (Vernon et al., 2015) or ear acupuncture (Murakami et al., 2017).

### Folk Remedies

Although traditional massage has been found to be effective in reducing migraine pain, it is not widely used and has not been reported to be as effective as lymphatic drainage massage (Happe et al., 2016). Echoing the findings of prior research, folk remedies were found in this study to be not as effective as pain management strategies backed by greater empirical support for efficacy (e.g., prescription or OTC medications, TCM). Specifically, traditional massage was rated as relatively low in effectiveness (42%) and was reported as having a short duration of efficacy (67% reported no effect or benefits lasting less than 1 day). However, the finding of a 50% effectiveness rate for gua sha therapy represents a new and consequential finding and suggests that gua sha warrants future study as a potentially effective traditional approach to migraine pain relief.

### Other Approaches to Migraine Management

In this study, sleep was found to be effective in alleviating migraine, with a very high effectiveness rating of 70%. This is consistent with the findings of previous research supporting the importance of sleep in migraine management. Insufficient sleep is known to be associated with an increased frequency of migraine attacks (Kim et al., 2017), and treating insomnia may reduce the frequency of migraine attacks (Sullivan et al., 2019). The findings of this study contribute to the literature further by indicating that the benefits of good sleep on migraine may persist for more than 1 day. This finding supports the need to further evaluate the efficacy of treatments that improve sleep quality in individuals with migraine, especially those reporting sleep problems, as a potential way to reduce migraine headache frequency and severity.

Drinking an adequate amount of coffee intermittently has also been cited as an effective strategy to alleviating migraine (Lee et al., 2016; Nehlig, 2016). Caffeine may also enhance the beneficial effects of analgesics on migraine (Nehlig, 2016), although in some cases, caffeine may aggravate migraine severity (Mostofsky et al., 2019). Consistent with the findings of previous studies, in this study, drinking coffee was found to be fairly effective in alleviating migraine (64%) over an extended duration (55% reported an effect greater than 1 day). Clinicians may find this information useful and incorporate it into recommendations for patients with migraine.

### Seeking Multiple Ways to More Effectively Manage Migraine

A new finding highlighted in this study is that a substantial subgroup of individuals with migraine (14% in this study) receives no significant effect from current pain management strategies. This underscores the need to identify additional and effective treatment options for people with migraine and to explore whether individuals in this subgroup are not using potentially effective management strategies.

Interestingly, and inconsistent with one of the study hypotheses, the participants who reported the greatest success in managing their migraine (i.e., perceived their means as “totally effective”) tended to use a single strategy only. Thus, rather than using a “shotgun” approach to target multiple causative factors, it may be that the most effective strategy is for individuals to identify a single approach that is most effective for their particular problem or situation if their headache responds well to just one treatment. Consistent with our hypothesis, those who reported the poorest results in managing their migraine (i.e., perceived their means as “totally ineffective”) were also more likely to report using a single treatment approach than their more-successful peers (Table 4). People may try to use the least medication possible, so one may expect a mild attack to be treated by one approach and a more severe attack by multiple approaches. Moreover, a second approach may be initiated later than the first approach, with the time gap affecting overall treatment efficacy. In pain management practice, multitherapy approaches have generally shown better effectiveness than single therapy approaches. For example, “acupuncture plus tui-na massage” showed better effect than “acupuncture only” in patients with migraine (Nie et al., 2019). In addition, “massage plus acupressure” showed to be relatively more effective than “massage only” or “acupressure only” on relieving labor pain (Gönenç & Terzioğlu, 2020). Thus, a multitherapy approach may be the future direction for research.

### Study Limitations

Several limitations should be considered when interpreting the findings of this study. First, the use of specific pain management approaches and their effectiveness were self-reported as recalled by the participants. Although the sample size was large and saturation seems to have been achieved, it is possible that management approaches used by some individuals with migraine were not reported here.

Second, a cross-sectional design was used in this study. Thus, it is not possible to draw conclusions regarding causal relationships among the study variables. Future researchers may use longitudinal designs and a headache diary to evaluate how changes in pain management approach are associated with and precede changes in headache activity. In addition, well-designed and adequately powered clinical trials will be necessary to confirm, for example, the superior effect of Western medicine (e.g., triptans) and TCM (e.g., acupuncture) over placebos in treating migraine pain.

### Summary and Conclusions

Despite its limitations, this study provides new and important knowledge regarding the use of pain management strategies in individuals with migraine. Our findings suggest the unmet needs in the pain management of migraine. As self-reported by the participants, all of whom were currently being treated by physicians, OTC medications (rather than prescription medications) were identified as most effective, with the highest prevalence and highest perceived efficacy. The findings also support the need to identify and evaluate the efficacy of additional treatment options for individuals with migraine so that all who are at risk of migraine may develop and use a management plan that is most effective for them.

## References

[bib1] Chang H.-Y. Jensen M. P. Yang C.-C., & Lai Y.-H. (2019). Migraine-Specific Quality of Life Questionnaire Chinese version 2.1 (MSQv2.1-C): Psychometric evaluation in patients with migraine. *Health and Quality of Life Outcomes*, 17(1), Article No. 108. 10.1186/s12955-019-1169-y 31234894 PMC6591995

[bib2] Dodick D. W. (2018). Migraine. *The Lancet*, 391(10127), 1315–1330. 10.1016/S0140-6736(18)30478-1 29523342

[bib3] Ertas M. Siva A. Dalkara T. Uzuner N. Dora B. Inan L. Idiman F. Sarica Y. Selçuki D. Sirin H. Oğuzhanoğlu A. Irkeç C. Ozmenoğlu M. Ozbenli T. Oztürk M. Saip S. Neyal M., & Zarifoğlu M., Turkish MIDAS Group. (2004). Validity and reliability of the Turkish Migraine Disability Assessment (MIDAS) questionnaire. *Headache*, 44(8), 786–793. 10.1111/j.1526-4610.2004.04146.x 15330825

[bib4] Gönenç I. M., & Terzioğlu F. (2020). Effects of massage and acupressure on relieving labor pain, reducing labor time, and increasing delivery satisfaction. *The Journal of Nursing Research*, 28(1), Article e68. 10.1097/jnr.0000000000000344 31524645

[bib5] Happe S. Peikert A. Siegert R., & Evers S. (2016). The efficacy of lymphatic drainage and traditional massage in the prophylaxis of migraine: A randomized, controlled parallel group study. *Neurological Sciences*, 37(10), 1627–1632. 10.1007/s10072-016-2645-3 27338942

[bib6] Hu X. H. Markson L. E. Lipton R. B. Stewart W. F., & Berger M. L. (1999). Burden of migraine in the United States: Disability and economic costs. *Archives of Internal Medicine*, 159(8), 813–818. 10.1001/archinte.159.8.813 10219926

[bib7] Hung P. H. Fuh J. L., & Wang S. J. (2006). Validity, reliability and application of the Taiwan version of the Migraine Disability Assessment questionnaire. *Journal of the Formosan Medical Association*, 105(7), 563–568. 10.1016/S0929-6646(09)60151-0 16877236

[bib8] Iigaya M. Sakai F. Kolodner K. B. Lipton R. B., & Stewart W. F. (2003). Reliability and validity of the Japanese Migraine Disability Assessment (MIDAS) Questionnaire. *Headache*, 43(4), 343–352. 10.1046/j.1526-4610.2003.03069.x 12656705

[bib9] Jiang Y. Bai P. Chen H. Zhang X.-Y. Tang X.-Y. Chen H.-Q. Hu Y.-Y. Wang X.-L. Li X.-Y. Li Y.-P., & Tian G.-H. (2018). The effect of acupuncture on the quality of life in patients with migraine: A systematic review and meta-analysis. *Frontiers in Pharmacology*, 9, Article 1190. 10.3389/fphar.2018.01190 30416444 PMC6212461

[bib10] Kim J. Cho S. J. Kim W. J. Yang K. I. Yun C. H., & Chu M. K. (2017). Insufficient sleep is prevalent among migraineurs: A population-based study. *The Journal of Headache and Pain*, 18(1), Article No. 51. 10.1186/s10194-017-0756-8 28455722 PMC5409905

[bib11] Ko H.-K. Chin C.-C., & Hsu M.-T. (2018). Moral distress model reconstructed using grounded theory. *The Journal of Nursing Research*, 26(1), 18–26. 10.1097/jnr.0000000000000189 29315204

[bib12] Lee M. J. Choi H. A. Choi H., & Chung C.-S. (2016). Caffeine discontinuation improves acute migraine treatment: A prospective clinic-based study. *The Journal of Headache and Pain*, 17(1), Article No. 71. 10.1186/s10194-016-0662-5 27492448 PMC4975726

[bib13] Lipton R. B. Munjal S. Buse D. C. Bennett A. Fanning K. M. Burstein R., & Reed M. L. (2017). Allodynia is associated with initial and sustained response to acute migraine treatment: Results from the american migraine prevalence and prevention study. *Headache*, 57(7), 1026–1040. 10.1111/head.13115 28603893

[bib14] Luo Y. Wang C.-Z. Sawadogo R. Tan T., & Yuan C.-S. (2020). Effects of herbal medicines on pain management. *American Journal of Chinese Medicine*, 48(1), 1–16. 10.1142/s0192415x20500019 32054304

[bib15] Macone A. E., & Perloff M. D. (2017). Triptans and migraine: Advances in use, administration, formulation, and development. *Expert Opinion on Pharmacotherapy*, 18(4), 387–397. 10.1080/14656566.2017.1288721 28129702

[bib16] Mayans L., & Walling A. (2018). Acute migraine headache: Treatment strategies. *American Family Physician*, 97(4), 243–251. 29671521

[bib17] Mostofsky E. Mittleman M. A. Buettner C. Li W., & Bertisch S. M. (2019). Prospective cohort study of caffeinated beverage intake as a potential trigger of headaches among migraineurs. *American Journal of Medicine*, 132(8), 984–991. 10.1016/j.amjmed.2019.02.015 PMC6744320 31402050

[bib18] Murakami M. Fox L., & Dijkers M. P. (2017). Ear acupuncture for immediate pain relief—A systematic review and meta-analysis of randomized controlled trials. *Pain Medicine*, 18(3), 551–564. 10.1093/pm/pnw215 28395101

[bib19] Nehlig A. (2016). Effects of coffee/caffeine on brain health and disease: What should I tell my patients? *Practical Neurology*, 16(2), 89–95. 10.1136/practneurol-2015-001162 26677204

[bib20] Nie L. Cheng J. Wen Y., & Li J. (2019). The effectiveness of acupuncture combined with tuina therapy in patients with migraine. *Complementary Medicine Research*, 26(3), 182–194. 10.1159/000496032 30893677

[bib21] Polit D. F., & Beck C. T. (2017). *Essentials of nursing research: Appraising evidence for nursing practice* (9th ed.). Lippincott Williams & Wilkins.

[bib22] Silberstein S. D., & Marmura M. J. (2015). Acute migraine treatment. *Headache*, 55(1), 1–2. 10.1111/head.12504 25600717

[bib23] Stewart W. F. Lipton R. B. Kolodner K. Liberman J., & Sawyer J. (1999). Reliability of the Migraine Disability Assessment score in a population-based sample of headache sufferers. *Cephalalgia*, 19(2), 107–114. 10.1046/j.1468-2982.1999.019002107.x 10214536

[bib24] Stovner L. J. Nichols E. Steiner T. J. Abd-Allah F. Abdelalim A. Al-Raddadi R. M. Ansha M. G. Barac A. Bensenor I. M. Doan L. P. Edessa D. Endres M. Foreman K. J. Gankpe F. G. Gopalkrishna G. Goulart A. C. Gupta R. Hankey G. J. Hay S. I., & Murray C. J. L. (2018). Global, regional, and national burden of migraine and tension-type headache, 1990–2016: A systematic analysis for the Global Burden of Disease Study 2016. *The Lancet Neurology*, 17(11), 954–976. 10.1016/S1474-4422(18)30322-3 30353868 PMC6191530

[bib25] Sullivan D. P. Martin P. R., & Boschen M. J. (2019). Psychological sleep interventions for migraine and tension-type headache: A systematic review and meta-analysis. *Scientific Reports*, 9(1), Article No. 6411. 10.1038/s41598-019-42785-8 31015531 PMC6478829

[bib26] Vernon H. Borody C. Harris G. Muir B. Goldin J., & Dinulos M. (2015). A randomized pragmatic clinical trial of chiropractic care for headaches with and without a self-acupressure pillow. *Journal of Manipulative and Physiological Therapeutics*, 38(9), 637–643. 10.1016/j.jmpt.2015.10.002 26548737

[bib27] Xiao Y. Yuan L. Liu Y. Sun X. Cheng J. Wang T. Li F. Luo R., & Zhao X. (2015). Traditional Chinese patent medicine for prophylactic treatment of migraine: A meta-analysis of randomized, double-blind, placebo-controlled trials. *European Journal of Neurology*, 22(2), 361–368. 10.1111/ene.12581 25346402

